# Synthesis and Antimicrobial Activity of New 5-(2-Thienyl)-1,2,4-triazoles and 5-(2-Thienyl)-1,3,4-oxadiazoles and Related Derivatives

**DOI:** 10.3390/molecules15010502

**Published:** 2010-01-22

**Authors:** Mohamed A. Al-Omar

**Affiliations:** Department of Pharmaceutical Chemistry, College of Pharmacy, King Saud University, Riyadh 11451, Saudi Arabia; E-Mail: malomar1@ksu.edu.sa

**Keywords:** 2-thienyl derivatives, 1,2,4-triazoles, 1,3,4-oxadiazoles, microwave irradiation, antimicrobial activity

## Abstract

New 5-(2-thienyl)-1,2,4-triazoles and 5-(2-thienyl)-1,3,4-oxadiazoles namely, *N*-[3-mercapto-5-(2-thienyl)-1,2,4-triazol-4-yl]-*N*'-arylthioureas **4a–e**, 2-arylamino-5-(2-thienyl)-1,2,4-triazolo[3,4-*b*][1,3,4]thiadiazoles **5a–e**, 3-arylaminomethyl-5-(2-thienyl)-1,3,4-oxadiazoline-2-thiones **7a–e**, 3-(*N*-substituted anilinomethyl)-5-(2-thienyl)-1,3,4-oxadiazoline-2-thiones **8a, b** and 3-(4-substituted-1-piperazinylmethyl)-5-(2-thienyl)-1,3,4-oxadiazoline-2-thiones **9a–f**, were prepared. The synthesized compounds were tested for *in vitro* activities against certain strains of Gram-positive and Gram-negative bacteria and the yeast-like pathogenic fungus *Candida albicans*. Compound **9a** displayed marked broad spectrum antibacterial activity, while compounds **4d**, **5e**, **7b**, **7c**, **7d**, **9b**, **9c** and **9d** were highly active against the tested Gram-positive bacteria. None of the synthesized compounds were proved to be significantly active against *Candida albicans*.

## 1. Introduction

In the past decades, the problem of multidrug resistant microorganisms has reached on alarming level around the world, and the synthesis of new anti-infective compounds has become an urgent need for the treatment of microbial infections. The 1,2,4-triazole nucleus has been incorporated into a wide variety of therapeutically important agents, mainly displaying antimicrobial [1,2,3,4,5] and anti-inflammatory activities [6,7,8,9]. In addition, several 1,3,4-oxadiazole derivatives were reported to exhibit good antimicrobial [10,11,12,13] and anti-inflammatory activities [13,14,15]. Moreover, the thiophene nucleus was proven to constitute the active part of several biologically active compounds [16,17,18,19,20]. In view of these findings, and in continuation to our interest in the synthesis and biological activities 1,2,4-triazoles and 1,3,4-oxadiazoles [7,12], the present investigation describes the synthesis as potential antimicrobial agents of new series of 1,2,4-triazoles and 1,3,4-oxadiazoles bearing 2-thienyl moieties.

## 2. Results and Discussion

### 2.1. Chemistry

**Scheme 1:**
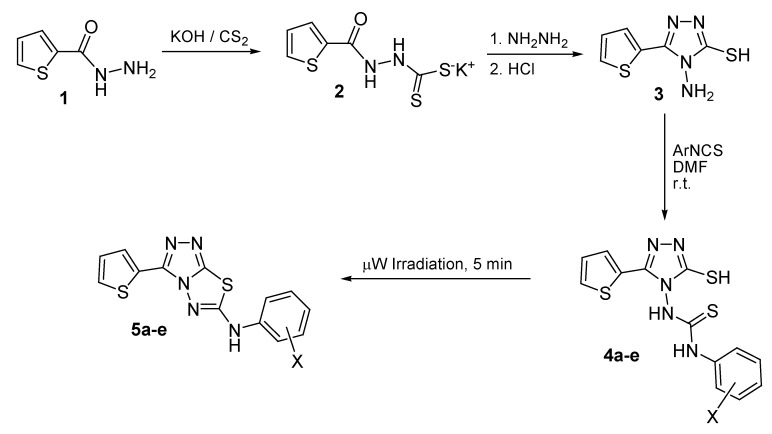
Synthetic Pathway for Compounds **4a–e** and **5a–e**.

4-Amino-5-mercapto-3-(2-thienyl)-1,2,4-triazole (**3**), required as starting material, was prepared *via* treatment of an ethanolic potassium hydroxide solution of thiophene-2-carbohydrazide (**1**) with carbon disulphide to yield the intermediate dithiocarbazate **2**, which was subsequently reacted with hydrazine to yield compound **3** [21]. It was reported that the nature of the reaction products of *N*-amino-1,2,4-triazoles with arylisothiocyanates was dependent on the reaction solvent and temperature [22,23]. Molina and Tárraga [22] reported the formation of the *N*,*N*'-disubstituted thiourea derivatives upon carrying out the reaction in *N*,*N*-dimethylformamide (DMF) at room temperature for 24 h and the formation of the 2,5-disubstituted-1,2,4-triazolo[3,4–*b*][1,3,4]thiadiazoles upon prolonged heating in DMF. On the other hand, carrying out the reaction under microwave irradiation in the absence of solvent yielded the corresponding 2,5-disubstituted-1,2,4-triazolo[3,4–*b*][1,3,4]thiadiazoles in good yield [23]. Thus, compound **3** was reacted with phenyl- or substituted phenylisothiocyanates in DMF at room temperature for 24 h to yield the corresponding *N*,*N*'-disubstituted thiourea derivatives **4a–e** in excellent yields (85–90%). Compounds **4a–e** were successfully dehydrosulphurized to the corresponding 2-arylamino-5-(2-thienyl)-1,2,4-triazolo[3,4–*b*][1,3,4]thiadiazole derivatives **5a–e** in 92%–95% yields *via* exposure to microwave irradiation for 5 min (Scheme 1, Table 1).

**Scheme 2:**
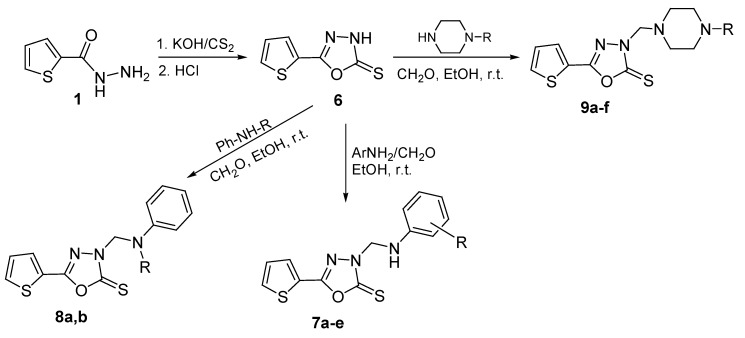
Synthetic Pathway for Compounds **7a–e**, **8a, b** and **9a–f**.

5-(2-Thienyl)-1,3,4-oxadiazoline-2-thione (**6**) was prepared *via* the reaction of the hydrazide **1** with carbon disulphide and potassium hydroxide, in ethanol, followed by acidification, following the previously reported methods [24,25,26]. Treatment of **6** with formaldehyde solution and primary aromatic amines, *N*-substituted anilines or *N*-substituted piperazines in ethanol at room temperature afforded good yields of the corresponding *N*-Mannich derivatives **7a–e**, **8a, b** and **9a–f**, respectively (Scheme **2**, Table 1). The structures of the synthesized compounds were confirmed by elemental analyses, IR, ^1^H-NMR, ^13^C-NMR, and mass spectral data.

### 2.2. Antimicrobial Testing

The newly synthesized compounds **4a–e**, **5a–e**, **7a–e**, **8a, b** and **9a–f** were tested for their *in vitro* antimicrobial activity against a panel of standard strains of the Gram-positive bacteria (*Staphylococcus aureus* IFO 3060 and *Bacillus subtilis* IFO 3007), the Gram-negative bacteria (*Escherichia coli* IFO 3301 and *Pseudomonas aeuroginosa* IFO 3448), and the yeast-like pathogenic fungus *Candida albicans* IFO 0583. The primary screening was carried out using the agar disc-diffusion method [27] using Müller-Hinton agar medium. Sterile filter paper discs (8 mm diameter) were moistened with the test compound solution in dimethylsulphoxide of specific concentration 200 μg/disc were carefully placed on the agar cultures plates that had been previously inoculated separately with the microorganisms. The plates were incubated at 37 °C, and the diameter of the growth inhibition zones were measured after 24 h in case of bacteria and 48 h in case of *Candida albicans*. The results of the preliminary antimicrobial testing of compounds **4a–e**, **5a–e**, **7a–e**, **8a, b** and **9a–f** (200 μg/disc), the antibacterial antibiotic Ampicillin trihydrate (100 μg/disc) and the antifungal drug clotrimazole (100 μg/disc) are shown in Table 2. The results revealed that the majority of the synthesized compounds showed varying degrees of inhibition against the tested microorganisms. In general, the best activity was displayed by compounds **4d**, **4e**, **5e**, **7b**, **7c**, **7d**, **9a**, **9b**, **9c** and **9d**, and the Gram-positive bacteria *Bacillus subtilis* is considered the most sensitive among the tested microorganisms. Compound **9a** showed high broad-spectrum inhibitory activity against the tested microorganisms, while compounds **4d**, **5e**, **7b**, **7c**, **7d**, **9b**, **9c** and **9d** were selectively active against the Gram-positive bacteria (inhibition zone >15 mm). Moderate (inhibition zone 12–15 mm) or weak (inhibition zone 10–12 mm) antibacterial activity was observed for compounds **4a**, **4b**, **4c**, **5b**, **5c**, **5d**, **7a** and **7e**. Meanwhile, compounds **5a**, **8a**, **8b**, **9e** and **9f** were inactive against the tested microorganisms. All the synthesized compounds were practically inactive against *Candida albicans*, compounds **9a**, **9c** and **9d** produced weak activity relative to the antifungal drug clotrimazole. The minimal inhibitory concentration (MIC) for the most active compounds **4b**, **4c**, **4d**, **4e**, **5e**, **7a**, **7b**, **7c**, **7d**, **9a**, **9b**. **9c** and **9d** against the same microorganism used in the primary screening was carried out using the microdilution susceptibility method in Müller-Hinton Broth and Sabouraud Liquid Medium [28]. The compounds, Ampicillin trihydrate and clotrimazole were dissolved in dimethylsulphoxide at concentration of 128 μg/mL. The twofold dilutions of the solution were prepared (128, 64, 32, …, 0.5 μg/mL). The microorganism suspensions at 10^6^ CFU/mL (colony forming unit/mL) concentrations were inoculated to the corresponding wells. The plates were incubated at 36 °C for 24 and 48 h for the bacteria and *Candida albicans*, respectively. The MIC values were determined as the lowest concentration that completely inhibited visible growth of the microorganism as detected by unaided eye. The MIC of the most active compounds, the antibacterial antibiotic ampicillin trihydrate and the antifungal drug clotrimazole which are shown in Table 3, were in accordance with the results obtained in the primary screening.

The structure-antibacterial activity relationship of the synthesized compounds revealed that the acyclic *N*,*N'*-disubstituted thiourea derivatives **4a–e** and their cyclic 2,5-disubstituted-1,2,4-triazolo[3,4–*b*][1,3,4]thiadiazoles analogues **5a–e** are almost of equal activity against the tested Gram-positive bacteria and almost inactive against the Gram-negative bacteria. The antibacterial activity of the oxadiazole *N*-Mannich derivatives **7a–e**, **8a, b** and **9a–f** indicated that antibacterial activity is mainly dependent on the aminomethyl substituents. The arylaminomethyl-1,3,4-oxadiazoline-2-thiones **7a–e** were almost active against both the Gram-positive and Gram-negative bacteria, while *N*-methyl or benzyl derivatives **8a, b** were completely inactive. Regarding the piperazine derivatives **9a–f**, the methyl, phenyl, 4-fluorophenyl and 2-trifluoromethylphenyl derivatives **9a**, **9b**, **9c** and **9d** were highly active against the Gram-positive bacteria and to lesser extent to the Gram-negative bacteria. On the other hand, replacement of the methyl or aryl substituents in compounds **9a**, **9b**, **9c** and **9d** with a 4-benzyl- or 2-trifluoromethylbenzyl moieties **9e** and **9f** dramatically reduced the antimicrobial activity. None of the synthesized compounds were found to be active against *Candida albicans*, and only the piperazine derivatives **9a**, **9c** and **9d** exhibited marginal activities.

## 3. Experimental 

### 3.1. General

All melting points (°C, uncorrected) were determined using a Gallenkamp melting point apparatus. Microwave irradiation was performed using an Akai MW-GB092MP (800 W) unmodified domestic microwave oven operated at 2450 MHz. Infra red spectra were recorded in KBr disc using Jasco FT/IR 460 Plus spectrometer, and expressed in wave number υ (cm^−1^). NMR spectra were obtained on a Bruker AC 500 Ultra Shield NMR spectrometer at 500 MHz for ^1^H and 125 MHz for ^13^C, the chemical shifts are expressed in δ (ppm) downfield from tetramethylsilane (TMS). Electron impact mass spectra were recorded on a Shimadzu GC–MS-QP 5000 instrument. Elemental analyses (C, H, N, S) were in full agreement with the proposed structures within ± 0.4% of the theoretical values. The bacterial strains and *Candida albicans* fungus were obtained from the Institute of fermentation of Osaka (IFO), Osaka, Japan. The reference drugs ampicillin trihydrate (CAS 7177-48-2) and clotrimazole (CAS 23593-75-1) were purchased from Sigma-Aldrich Chemie GmbH (Taufkirchen, Germany).

### 3.2. N-[3-Mercapto-5-(2-thienyl)-1,2,4-triazol-4-yl]-N'-arylthioureas ***4a–e***

The appropriate arylisothio-cyanate (2 mmol) was added to a solution of 4-amino-3-mercapto-5-(2-thienyl)-1,2,4-triazole (**3**, 0.4 g, 2 mmol) in dry DMF (8 mL), and the solution was stirred at room temperature for 24 h. Water (20 mL) was then added and the mixture was stirred for 20 min. The separated precipitate was filtered, washed with water and crystallized from ethanol.

**4a**: IR, υ (cm^−1^): 3,305 (NH), 3,021 (Ar-CH), 1,604, 1,452 (C=N), 1,374 (C=S); ^1^H-NMR (DMSO-d_6_): δ 6.78–7.03 (m, 3H, Ar-H), 7.15–7.32 (m, 5H, Ar-H & thiophene-H), 10.01 (s, 1H, NH), 10.62 (s, 1H, NH), 13.88 (s, 1H, SH); ^13^C-NMR: δ 123.52, 125.44, 127.01, 127.32, 127.89, 129.55, 136.42, 142.75 (Ar-C & thiophene-C), 143.53 (triazole C-5), 159.50 (triazole C-3), 169.54 (C=S); MS, *m/z* (rel. int.): 333 (M+, 6), 301 (9), 182 (54), 92 (88), 77, (72), 58 (100).

**4b**: IR, υ (cm^−1^): 3308 (NH), 3022 (Ar-CH), 1610, 1452 (C=N), 1322 (C=S). ^1^H NMR (DMSO-d_6_): δ 6.89–7.98 (m, 7H, Ar-H & Thiophene-H), 10.16 (s, 1H, NH), 10.95 (s, 1H, NH), 13.95 (s, 1H, SH). ^13^C NMR: δ 110.78, 114.76, 122.0, 125.50, 126.75, 127.95, 130.62, 136.20, 141.43, 160.25 (Ar-C & Thiophene-C), 146.53 (triazole C-5), 164.44 (triazole C-3), 172.82 (C=S). MS, *m/z* (Rel. Int.): 351 (M+, 1), 319 (3), 182 (61), 154 (9), 111 (77), 57 (100).

**4c**: IR, υ (cm^−1^): 3331 (NH), 3022 (Ar-CH), 1610, 1460 (C=N), 1371 (C=S). ^1^H NMR (DMSO-d_6_): δ 6.81–7.75 (m, 7H, Ar-H & Thiophene-H), 10.15 (s, 1H, NH), 10.97 (s, 1H, NH), 14.05 (s, 1H, SH). ^13^C NMR: δ 113.65, 123.05, 126.98, 127.90, 128.88, 132.24, 142.35, 159.88 (Ar-C & Thiophene-C), 147.90 (triazole C-5), 168.01 (triazole C-3), 171.98 (C=S). MS, *m/z* (Rel. Int.): 351 (M+, 4), 319 (11), 182 (69), 111 (71), 57 (100).

**4d**: IR, υ (cm^−1^): 3310 (NH), 3025 (Ar-CH), 1598, 1450 (C=N), 1377 (C=S). ^1^H NMR (DMSO-d_6_): δ 7.21–7.42 (m, 5H, Ar-H & Thiophene-H), 7.63 (d, 2H, Ar-H, *J* = 8.2 Hz), 10.01 (s, 1H, NH), 10.88 (s, 1H, NH), 13.88 (s, 1H, SH). ^13^C NMR: δ 124.11, 127.60, 127.96, 129.05, 129.54, 136.10, 143.25 (Ar-C & Thiophene-C), 148.88 (triazole C-5), 167.21 (triazole C-3), 168.33 (C=S). MS, *m/z* (Rel. Int.): 369 (M^+^ +2, 2), 367 (M+, 5), 335 (6), 333 (13), 182 (44), 126 (22), 111 (64), 57 (100).

**4e**: IR, υ (cm^−1^): 3343 (NH), 3010 (Ar-CH), 1600, 1442 (C=N), 1388 (C=S). ^1^H NMR (DMSO-d_6_): δ 7.22–7.98 (m, 7H, Ar-H & Thiophene-H), 10.80 (s, 1H, NH), 11.0 (s, 1H, NH), 14.06 (s, 1H, SH). ^13^C NMR: δ 121.07, 126.10, 128.43, 129.59, 130.12, 131.72, 135.29, 143.65 (Ar-C & Thiophene-C), 146.33 (triazole C-5), 167.29 (triazole C-3), 168.31 (C=S). MS, *m/z* (Rel. Int.): 413 (M^+^ +2, 5), 411 (M^+^, 4), 377 (8), 379 (10), 182 (23), 171 (25), 169 (25), 57 (100).

### 3.3. 2-Arylamino-5-(2-thienyl)-1,2,4-triazolo[3,4-b][1,3,4]thiadiazoles ***5a–e***

The appropriate *N*,*N*'-disubstituted thiourea **4a–c** (2 mmol) was placed in 50 mL open round bottom flask, and the mixture was irradiated in the microwave oven for 5 min. at 454 W (58%). On cooling, chloroform (10 mL) was added and the reaction mixture was stirred for 5 min., then filtered and the filtrate was evaporated *in vacuo*. The crude product was crystallized to yield the desired compounds **5a–e**.

**5a**: IR, υ (cm^−1^): 3221 (NH), 3001 (Ar-CH), 1620, 1459 (C=N). ^1^H NMR (CDCl_3_): δ 6.44 (s, 1H, NH), 6.95–7.05 (m, 3H, Ar-H), 7.22–7.34 (m, 5H, Ar-H and Thiophene-H). ^13^C NMR: δ 117.50, 120.05, 125.44, 127.62, 129.82, 139.92, 142.45, 144.56 (Ar-C & Thiophene-C), 148.70 (C-8), 151.10 (C-5), 172.15 (C-2). MS, *m/z* (Rel. Int.): 300 (M^+^ +1, 2), 299 (M^+^, 24), 223 (26), 91 (65), 77 (100), 57 (13).

**5b**: IR, υ (cm^−1^): 3245 (NH), 3010 (Ar-CH), 1654, 1462 (C=N). ^1^H NMR (CDCl_3_): δ 6.93 (s, 1H, NH), 6.92 (s, 1H, Ar-H), 7.02–7.62 (m, 6H, Ar-H and Thiophene-H). ^13^C NMR: δ 105.66, 108.78, 113.23, 124.50, 127.90, 128.05, 130.85, 142.22, 144.60, 161.09 (Ar-C & Thiophene-C), 151.44 (C-8), 154.40 (C-5), 177.80 (C-2). MS, *m/z* (Rel. Int.): 317 (M+, 26), 207 (11), 110 (34), 96 (12), 57 (100).

**5c**: IR, υ (cm^−1^): 3241 (NH), 3012 (Ar-CH), 1623, 1466 (C=N). ^1^H NMR (CDCl_3_): δ 6.67–6.97 (m, 3H, Ar-H & NH), 7.15–7.62 (m, 5H, Ar-H and Thiophene-H). ^13^C NMR: δ 114.50, 119.02, 124.94, 127.24, 128.05, 136.65, 142.75, 150.76 (Ar-C & Thiophene-C), 151.98 (C-8), 156.34 (C-5), 178.05 (C-2). MS, *m/z* (Rel. Int.): 317 (M+, 33), 207 (23), 110 (42), 96 (9), 57 (100).

**5d**: IR, υ (cm^−1^): 3259 (NH), 3044 (Ar-CH), 1626, 1461 (C=N). ^1^H NMR (CDCl_3_): δ 6.44 (s, 1H, NH), 7.08 (d, 2H, Ar-H, *J* = 8.0 Hz), 7.45–7.63 (m, 5H, Ar-H & Thiophene-H). ^13^C NMR: δ 115.44, 122.90, 124.0, 127.10, 127.65, 130.22, 136.55, 141.80 (Ar-C & Thiophene-C), 149.51 (C-8), 155.25 (C-5), 174.85 (C-2). MS, *m/z* (Rel. Int.): 335 (M^+^ +2, 7), 333 (M^+^, 24), 207 (35), 126 (19), 113 (7), 111 (20), 57 (100).

**5e**: IR, υ (cm^−1^): 3251 (NH), 3014 (Ar-CH), 1661, 1465 (C=N). ^1^H NMR (CDCl_3_): δ 6.52 (s, 1H, NH), 7.16 (d, 2H, Ar-H, *J* = 8.2 Hz), 7.33–7.68 (m, 5H, Ar-H & Thiophene-H). ^13^C NMR: δ 112.23, 118.24, 124.90, 127.02, 127.65, 132.45, 142.05, 144.50 (Ar-C & Thiophene-C), 146.60 (C-8), 153.90 (C-5), 172.82 (C-2). MS, *m/z* (Rel. Int.): 379 (M^+^ +2, 51), 377 (M^+^, 62), 207 (52), 172 (42), 170 (41), 158 (8), 156 (10), 57 (100).

### 3.4. 3-Arylaminomethyl-5-(2-thienyl)-1,3,4-oxadiazoline-2-thiones ***7a–e***, 3-(N-substituted anilinomethyl)- 5-(2-thienyl)-1,3,4-oxadiazoline-2-thiones ***8a**,**b*** and 3-(4-substituted-1-piperazinylmethyl)-5-(2-thienyl)-1,3,4-oxadiazoline-2-thiones ***9a–f***

Τhe appropriate primary aromatic amine, *N*-substituted aniline or 1-substituted piperazine (2 mmol) and 37% formaldehyde solution (0.5 mL) were added to a solution of 5-(2-thienyl)-1,3,4-oxadiazoline-2-thione (**6**, 0.37 g, 2 mmol) in ethanol (8 mL), and the mixture was stirred at room temperature for 2 h and allowed to stand overnight. The separated precipitate was filtered, washed with cold ethanol, dried, and crystallized.

**7a**: IR, υ (cm^−1^): 3336 (NH), 3034 (Ar-CH), 2898 (CH_2_), 1604, 1466 (C=N) 1258 (C-O-C). ^1^H NMR (CDCl_3_): δ 5.42 (s, 1H, NH), 5.64 (s, 2H, CH_2_), 6.80–7.50 (m, 5H, Ar-H & Thiophene-H), 7.53 (d, 1H, Thiophene-H, *J* = 5.0 Hz), 7.71 (d, 1H, Thiophene-H, *J* = 5.0 Hz). ^13^C NMR: δ 58.92 (CH_2_), 114.90, 116.21, 118.82, 123.43, 125.55, 127.23, 128.31, 130.80, 140.10, 154.95 (Ar-C & Thiophene-C), 156.90 (C=N), 177.30 (C=S). MS *m/z* (Rel. Int.): 307 (M^+^, 1), 207 (14), 183 (90), 105 (22), 57 (100).

**7b**: IR, υ (cm^−1^): 3342 (NH), 3052 (Ar-CH), 2912 (CH_2_), 1612, 1458 (C=N) 1266 (C-O-C). ^1^H NMR (CDCl_3_): δ 5.47 (d, ^1^H, NH, *J* = 8.5 HZ), 5.69 (s, 2H, CH_2_), 6.86–7.89 (m, 5H, Ar-H & Thiophene-H), 7.56 (d, 1H, Thiophene-H, *J* = 4.5 Hz), 7.69 (d, 1H, Thiophene-H, *J* = 4.5 Hz). ^13^C NMR: δ 58.92 (CH_2_), 115.49, 117.12, 123.43, 128.31, 130.80, 140.10, 155.24 (Ar-C & Thiophene-C), 158.74 (C=N), 176.25 (C=S). MS *m/z* (Rel. Int.): 307 (M^+^, 2), 207 (65), 183 (88), 105 (78), 57 (100).

**7c**: IR, υ (cm^−1^): 3349 (NH), 3040 (Ar-CH), 2891 (CH_2_), 1619, 1460 (C=N) 1265 (C-O-C). ^1^H NMR (CDCl_3_): δ 5.40 (s, 1H, NH), 5.65 (s, 2H, CH_2_), 6.65 (d, 2H, Ar-H, *J* = 8.5 Hz), 7.22 (d, 2H, Ar-H, *J* = 8.5 Hz), 7.43 (t, 1H, Thiophene-H, *J* = 5.0 Hz), 7.56 (d, 1H, Thiophene-H, *J* = 5.0 Hz), 7.69 (d, 1H, Thiophene-H, *J* = 5.0 Hz). ^13^C NMR: δ 58.92 (CH_2_), 115.49, 117.12, 123.43, 128.31, 129.95, 131.02, 134.23, 136.42 (Ar-C & Thiophene-C), 156.44 (C=N), 176.98 (C=S). MS *m/z* (Rel. Int.): 323 (M^+^, 1), 217 (25), 183 (85), 125 (88), 127 (23), 57 (100).

**7d**: IR, υ (cm^−1^): 3360 (NH), 3043 (Ar-CH), 2880 (CH_2_), 1615, 1451 (C=N) 1254 (C-O-C). ^1^H NMR (CDCl_3_): δ 4.45 (s, 1H, NH), 5.58 (s, 2H, CH_2_), 6.95–7.07 (m, 2H, Ar-H), 7.20–7.23 (m, 1H, Ar-H), 7.45–7.60 (m, 3H, Ar-H & Thiophene-H), 7.72 (d, 1H, Thiophene-H, *J* = 5.0 Hz). ^13^C NMR: δ 57.88 (CH_2_), 113.61, 121.38, 121.90, 122.16, 123.37, 125.63, 126.88, 128.39, 130.99, 131.23, 146.85 (Ar-C, CF_3_ & Thiophene-C), 156.02 (C=N), 176.04 (C=S). MS *m/z* (Rel. Int.): 357 (M^+^, 0.5), 183 (56), 174 (8), 145 (75), 57 (100).

**7e**: IR, υ (cm^−1^): 3322 (NH), 3065 (Ar-CH), 2887 (CH_2_), 1622, 1460 (C=N) 1249 (C-O-C). ^1^H NMR (CDCl_3_): δ 4.44 (s, 1H, NH), 5.62 (s, 2H, CH_2_), 6.87–7.0 (m, 2H, Ar-H), 7.17–7.52 (m, 4H, Ar-H & Thiophene-H), 7.68 (d, 1H, Thiophene-H, *J* = 5.0 Hz). ^13^C NMR: δ 57.54 (CH_2_), 108.55, 112.36, 120.24, 121.64, 122.17, 124.25, 126.82, 126.98, 128.0, 131.05, 132.25, 143.98 (Ar-C, CF_3_ & Thiophene-C), 157.08 (C=N), 177.20 (C=S). MS *m/z* (Rel. Int.): 357 (M^+^, 0.5), 183 (21), 174 (6), 145 (64), 57 (100).

**8a**: IR, υ (cm^−1^): 3322 (NH), 3021 (Ar-CH), 2910, 2852 (CH_2_, CH_3_), 1618, 1450 (C=N), 1371 (C=S), 1251 (C-O-C). ^1^H NMR (CDCl_3_): δ 3.28 (s, 3H, CH_3_), 5.64 (d, 2H, NCH_2_N), 6.83–7.36 (m, 6H, Ar-H & Thiophene-H), 7.55 (d, 1H, Thiophene-H, *J* = 5.0 Hz), 7.70 (d, 1H, Thiophene-H, *J* = 5.0 Hz). ^13^C NMR: δ 39.54 (CH_3_), 66.80 (CH_2_), 113.72m 119.12, 123.65, 128.30, 129.36, 130.36, 131.07, 146.69 (Ar-C & Thiophene-C), 155.89 (C=N), 176.32 (C=S). MS *m/z* (Rel. Int.): 303 (M^+^, 1), 282 (22), 193 (65), 97 (91), 57 (100).

**8b**: IR, υ (cm^−1^): 3325 (NH), 3012, 3027 (Ar-CH), 2913, 2905 (CH_2_), 1604, 1451 (C=N), 1370 (C=S), 1259 (C-O-C). ^1^H NMR (CDCl_3_): δ 4.99 (s, 2H, CH_2_Ph), 5.77 (s, 2H, NCH_2_N), 6.87–6.90 (m, 1H, Ar-H), 7.05–7.07 (m, 2H, Ar-H), 7.11–7.18 (m, 1H, Ar-H), 7.25–7.41 (m, 7H, Ar-H & Thiophene-H), 7.56 (d, 1H, Thiophene-H, *J* = 5.0 Hz), 7.69 (d, 1H, Thiophene-H, *J* = 5.0 Hz). ^13^C NMR: δ 55.05 (***C***H_2_Ph), 56.41 (N***C***H_2_N), 114.26, 119.43, 123.69, 126.67, 127.20, 128.28, 128.74, 129.39, 130.77, 131.0, 137.87, 146.44 (Ar-C & Thiophene-C), 155.93 (C=N), 176.31 (C=S). MS *m/z* (Rel. Int.): 397 (M^+^, 1), 341 (12), 282 (27), 159 (75), 92 (98), 57 (100).

**9a**: IR, υ (cm^−1^): 3330 (NH), 3004 (Ar-CH), 2918, 2810 (CH_2_, CH_3_), 1588, 1441 (C=N), 1381 (C=S), 1261 (C-O-C). ^1^H NMR (CDCl_3_): δ 2.33 (s, 3H, CH_3_), 2.56–2.62 (m, 4H, Piperazine-H), 2.96–3.02 (m, 4H, Piperazine-H), 5.19 (s, 2H, CH_2_), 7.36–7.44 (m, 2H, Thiophene-H), 7.71 (d, 1H, Thiophene-H, *J* = 5.0 Hz). ^13^C NMR: δ 43.62 (CH_3_), 52.20, 54.42 (Piperazine-C), 67.71 (CH_2_), 126.42, 127.98, 128.36, 130.85 (Thiophene-C), 157.28 (CH=N), 176.44 (C=S). MS *m/z* (Rel. Int.): 296 (M^+^, 0.5), 183 (35), 113 (22), 98 (28, 57 (100).

**9b**: IR, υ (cm^−1^): 3333 (NH), 3023 (Ar-CH), 2921, 2816 (CH_2_), 1592, 1445 (C=N), 1380 (C=S), 1266 (C-O-C). ^1^H NMR (CDCl_3_): δ 3.0–3.3.08 (m, 4H, Piperazine-H), 3.18–3.26 (m, 4H, Piperazine-H), 5.14 (s, 2H, CH_2_), 6.87–6.96 (m, 3H, Ar-H), 7.18 (t, 1H, Thiophene-H, *J* = 4.3 Hz), 7.22–7.32 (m. 2H, Ar-H), 7.59 (d, 1H, Thiophene-H, *J* = 4.5 Hz), 7.75 (d, 1H, Thiophene-H, *J* = 4.5 Hz). ^13^C NMR: δ 49.37, 50.30 (Piperazine-C), 70.40 (CH_2_), 116.45, 120.06, 123.67, 128.33, 129.14, 130.77, 130.98, 151.26 (Ar-C & Thiophene-C), 155.47 (C=N), 177.76 (C=S). MS *m/z* (Rel. Int.): 358 (M^+^, 1), 341 (4), 183 (14), 77 (88), 57 (100).

**9c**: IR, υ (cm^−1^): 3336 (NH), 3023 (Ar-CH), 2909, 2875, 2798 (CH_2_), 1601, 1456 (C=N), 1378 (C=S), 1260 (C-O-C). ^1^H NMR (CDCl_3_): δ 3.02–3.10 (m, 4H, Piperazine-H), 3.18 (s, 4H, Piperazine-H), 5.24 (s, 2H, CH_2_), 6.96 (d, 2H, Ar-H, *J* = 7.5 Hz), 7.35–7.41 (m, 3H, Ar-H & Thiophene-H), 7.52 (d, 1H, Thiophene-H, *J* = 5.0 Hz), 7.70 (d, 1H, Thiophene-H, *J* = 5.0 Hz). ^13^C NMR: 50.12, 51.60 (Piperazine-C), 69.95 (CH_2_), 115.23, 117.23, 125.28, 127.43, 127.46, 129.37, 145.01, 149.606 (Ar-C), 157.0 (CH=N), 177.49 (C=S). MS *m/z* (Rel. Int.): 376 (M^+^, 2), 183 (34), 95 (87), 57 (100).

**9d**: IR, υ (cm^−1^): 3338 (NH), 3022 (Ar-CH), 2911, 2849 (CH_2_), 1589, 1438 (C=N), 1378 (C=S), 1267 (C-O-C). ^1^H NMR (CDCl_3_): δ 3.01–3.10 (m, 4H, Piperazine-H), 3.23–3.31 (m, 4H, Piperazine-H), 5.14 (s, 2H, CH_2_), 7.05–7.12 (m, 3H, Ar-H), 7.18–7.20 (m, 1H, Ar-H), 7.34 (t, 1H, Thiophene-H, *J* = 7.0 Hz), 7.60 (d, 1H, Thiophene-H, *J* = 7.0 Hz), 7.75 (d, 1H, Thiophene-H, *J* = 7.0 Hz). ^13^C NMR: δ 48.84, 50.11 (Piperazine-C), 70.29 (CH_2_), 112.52, 116.16, 119.05, 123.19, 123.26, 125.35, 128.34, 129.58, 130.80, 131.0, 151.35 (Ar-C, CF_3_ & Thiophene-C), 155.52 (C=N), 177.77 (C=S). MS *m/z* (Rel. Int.): 426 (M^+^, 1), 340 (43), 282 (32), 183 (6), 145 (22), 83 (11), 57 (100).

**9e**: IR, υ (cm^−1^): 3344 (NH), 3023 (Ar-CH), 2921, 2841 (CH_2_), 1601, 1454 (C=N), 1372 (C=S), 1258 (C-O-C). ^1^H NMR (CDCl_3_): δ 2.56 (s, 4H, Piperazine-H), 2.95 (s, 4H, Piperazine-H), 3.58 (s, 2H, CH_2_Ar), 5.12 (s, 2H, NCH_2_N), 7.13–7.44 (m, 6H, Ar-H & Thiophene-H), 7.52 (d, 1H, Thiophene-H, *J* = 7.0 Hz), 7.77 (d, 1H, Thiophene-H, *J* = 7.0 Hz). ^13^C NMR: δ 50.18, 52.90 (Piperazine-C), 63.02 (CH_2_Ar), 70.41 (N***C***H_2_N), 123.68, 127.16, 128.26, 128.33, 129.21, 130.72, 130.96, 137.84 (Ar-C, Thiophene-C), 155.36 (C=N), 177.69 (C=S). MS *m/z* (Rel. Int.): 372 (M^+^, 1), 281 (40), 183 (9), 92 (66), 77 (65), 57 (100).

**9f**: IR, υ (cm^−1^): 3341 (NH), 3014 (Ar-CH), 2922, 2781 (CH_2_), 1604, 1457 (C=N), 1372 (C=S), 1251 (C-O-C). ^1^H NMR (CDCl_3_): δ 2.54 (s, 4H, Piperazine-H), 2.88–2.96 (m, 4H, Piperazine-H), 3.67 (s, 2H, CH_2_Ar), 5.08 (s, 2H, NCH_2_N), 7.19–7.20 (m, 1H, Ar-H), 7.31–7.34 (m, 1H, Ar-H), 7.48 (t, 1H, Thiophene-H, *J* = 7.0 Hz), 7.60–7.63 (m, 2H, Ar-H & Thiophene-H), 7.75–7.77 (m, 2H, Ar-H & Thiophene-H). ^13^C NMR: δ 50.36, 53.04 (Piperazine-C), 58.07 (CH_2_Ar), 70.56 (N***C***H_2_N), 123.80, 125.66, 125.71, 126.74, 128.30, 128.48, 128.72, 130.29, 130.68, 130.86, 137.61 (Ar-C, CF_3_ & Thiophene-C), 155.40 (C=N), 177.78 (C=S). MS *m/z* (Rel. Int.): 440 (M^+^, 0.5), 357 (41), 281 (26), 183 (15), 159 (65), 83 (26), 57 (100).

## 4. Conclusions

In this study, new series of 1,2,4-triazoles and 1,3,4-oxadiazoles carrying 2-thienyl moieties as potential antimicrobial agents. The new derivatives were characterized by elemental analyses, IR, ^1^H NMR, ^13^C NMR, and mass spectral data. The new derivatives were tested for *in vitro* antimicrobial activities against a panel of Gram-positive bacteria (*Staphylococcus aureus* IFO 3060 and *Bacillus subtilis* IFO 3007), the Gram-negative bacteria (*Escherichia coli* IFO 3301 and *Pseudomonas aeuroginosa* IFO 3448) and the yeast-like pathogenic fungus *Candida albicans* IFO 0583. Compound **9a** displayed marked broad spectrum antibacterial activity, while compounds **4d**, **5e**, **7b**, **7c**, **7d**, **9b**, **9c** and **9d** were highly active against the tested Gram-positive bacteria. None of the synthesized compounds were proved to be significantly active against *Candida albicans*. Though, the mechanism of the antibacterial activity needs further investigations, which are in progress.

## Figures and Tables

**Table 1:** Crystallization solvents, melting points, yield percentages, molecular formulae, and molecular weights of compounds **4a–e**, **5a–e**, **7a–e**, **8a, b** and **9a–f**.

Comp. No.	X/R	Cryst. Solvent	M.p. (°C)	Yield (%)	Mol. Formula (Mol. Wt.)
**4a**	H	EtOH/H_2_O	205-7 (Dec.)	85	C_13_H_11_N_5_S_3_ (333.45)
**4b**	3-F	EtOH/H_2_O	169-71 (Dec.)	89	C_13_H_10_FN_5_S_3_ (351.45)
**4c**	4-F	EtOH/H_2_O	174-6 (Dec.)	85	C_13_H_10_FN_5_S_3_ (351.45)
**4d**	4-Cl	EtOH/H_2_O	169-71 (Dec.)	88	C_13_H_10_ClN_5_S_3_ (367.9)
**4e**	4-Br	EtOH	210-2 (Dec.)	90	C_13_H_10_BrN_5_S_3_ (412.35)
**5a**	H	EtOH	292-4	94	C_13_H_9_N_5_S_2_ (299.37)
**5b**	3-F	EtOH	266-8	90	C_13_H_8_FN_5_S_2_ (317.36)
**5c**	4-F	EtOH	275-277	90	C_13_H_8_FN_5_S_2_ (317.36)
**5d**	4-Cl	EtOH	>300	95	C_13_H_8_ClN_5_S_2_ (333.82)
**5e**	4-Br	EtOH/CHCl_3_	>300	95	C_13_H_8_BrN_5_S_2_ (378.27)
**7a**	2-F	EtOH	107-9	72	C_13_H_10_FN_3_OS_2_ (307.37)
**7b**	4-F	EtOH	123-5	81	C_13_H_10_FN_3_OS_2_ (307.37)
**7c**	4-Cl	EtOH	175-7	85	C_13_H_10_ClN_3_OS_2_ (323.82)
**7d**	2-CF_3_	EtOH	133-135	68	C_14_H_10_F_3_N_3_OS_2_ (357.37)
**7e**	3-CF_3_	EtOH	108-10	75	C_14_H_10_F_3_N_3_OS_2_ (357.37)
**8a**	CH_3_	EtOH	112-4	92	C_14_H_13_N_3_OS_2_ (303.4)
**8b**	C_6_H_5_CH_2_	EtOH	120-2	90	C_20_H_17_N_3_OS_2_ (379.5)
**9a**	CH_3_	EtOH/H_2_O	96-8	56	C_12_H_16_N_4_OS_2_ (296.41)
**9b**	C_6_H_5_	EtOH	131-3	70	C_17_H_18_N_4_OS_2_ (358.48)
**9c**	4-FC_6_H_4_	EtOH	120-2	77	C_17_H_17_N_4_OFS_2_ (376.46)
**9d**	2-CF_3_C_6_H_4_	EtOH	141-3	72	C_18_H_17_F_3_N_4_OS_2_ (426.48)
**9e**	C_6_H_5_CH_2_	EtOH	101-3	65	C_18_H_20_N_4_OS_2_ (372.51)
**9f**	2-CF_3_C_6_H_2_CH_2_	EtOH	125-7	72	C_19_H_19_N_4_OF_3_S_2_ (440.50)

**Table 2:** Antimicrobial activity of compounds **4a–e**, **5a–e**, **7a–e**, **8a, b** and **9a–f** (200 μg/8 mm disc), the broad spectrum antibacterial drug ampicillin (100 μg/8 mm disc) and the antifungal drug clotrimazole (100 μg/8 mm disc) against *Staphylococcus aureus* IFO 3060 (*SA*), *Bacillus subtilis* IFO 3007 (*BS*), *Escherichia coli* IFO 3301 (*EC*), *Pseudomonas aeuroginosa* IFO 3448 (*PA*), and *Candida albicans* IFO 0583 (*CA*).

Comp. No.	Diameter of Growth Inhibition Zone (mm)*				
	*SA*	*BS*	*EC*	*PA*	*CA*
**4a**	12	14	–	–	–
**4b**	12	16	–	–	–
**4c**	14	16	–	–	–
**4d**	17	19	–	–	–
**4e**	16	16	12	–	–
**5a**	–	–	–	–	–
**5b**	10	12	–	–	–
**5c**	12	12	–	–	–
**5d**	13	13	–	–	–
**5e**	16	17	14	–	–
**7a**	14	18	12	12	–
**7b**	16	18	14	14	–
**7c**	18	18	14	12	–
**7d**	17	18	12	12	–
**7e**	14	15	12	–	–
**8a**	–	–	–	–	–
**8b**	–	–	–	–	–
**9a**	18	18	16	17	12
**9b**	16	18	12	10	–
**9c**	17	18	15	12	12
**9d**	18	16	16	14	12
**9e**	–	–	–	–	–
**9f**	–	–	–	–	–
**Ampicillin**	19	18	16	15	NT
**Clotrimazole**	NT	NT	NT	NT	21

* (–): Inactive, no inhibition zone. (NT): Not tested.

**Table 3:** The minimal inhibitory concentrations (MIC, μg/mL) of compounds **4b**, **4c**, **4d**, **4e**, **5e**, **7a**, **7b**, **7c**, **7d**, **9a**, **9b**. **9c** and **9d**, the broad spectrum antibacterial drug Ampicillin and the antifungal drug Clotrimazole against *Staphylococcus aureus* IFO 3060 (*SA*), *Bacillus subtilis* IFO 3007 (*BS*), *Escherichia coli* IFO 3301 (*EC*), *Pseudomonas aeuroginosa* IFO 3448 (*PA*), and *Candida albicans* IFO 0583 (*CA*).

Comp. No.	Minimal Inhibitory Concentration (MIC, μg/mL)*				
	*SA*	*BS*	*EC*	*PA*	*CA*
**4b**	ND	4	ND	ND	ND
**4c**	ND	4	ND	ND	ND
**4d**	4	1	ND	ND	ND
**4e**	8	4	ND	ND	ND
**5e**	ND	4	ND	ND	ND
**7a**	ND	2	ND	ND	ND
**7b**	4	2	ND	ND	ND
**7c**	4	2	ND	ND	ND
**7d**	2	1	ND	ND	ND
**9a**	2	2	4	2	ND
**9b**	4	2	ND	ND	ND
**9c**	2	2	ND	ND	ND
**9d**	2	4	4	ND	ND
**Ampicillin**	1	0.5	2	2	ND
**Clotrimazole**	ND	ND	ND	ND	2

* ND: Not determined.
